# Genome-Wide Association Study to Identify Soybean Lodging Resistance Loci and Candidate Genes

**DOI:** 10.3390/ijms26094446

**Published:** 2025-05-07

**Authors:** Zicong Liang, Nianhua Qi, Ruoning Li, Ruijia Gao, Junxia Huang, Wei Zhao, Huijun Zhang, Haiying Wang, Xue Ao, Xingdong Yao, Futi Xie

**Affiliations:** Soybean Research Institute, Shenyang Agricultural University, Shenyang 110866, China; jiuweiconghua@163.com (Z.L.); qinianhua@163.com (N.Q.); liruoningsoy@163.com (R.L.); gaoruijia0301@163.com (R.G.); hjx11150906@163.com (J.H.); shu375053911@163.com (W.Z.); 1991500012@syau.edu.cn (H.Z.); wanghy99@syau.edu.cn (H.W.); a2009syau@syau.edu.cn (X.A.)

**Keywords:** soybean, genome-wide association, lodging, single nucleotide polymorphism (SNP), candidate gene

## Abstract

High-density planting is crucial for maximizing the genetic potential of soybean cultivars to achieve higher yields. However, increasing the planting density can lead to the risk of plant lodging. Therefore, the identification of gene loci associated with lodging resistance is considered critical for the development of high-yielding, lodging-resistant soybean cultivars. In this study, 338 natural soybean accessions from the similar latitude were used to identify candidate genes associated with lodging resistance. Based on 9,400,987 SNPs, the soybean population was classified into three subpopulations. Genome-wide association analysis revealed that under planting densities of 300,000 and 150,000 plants/ha, a total of 20 significant SNPs were repeatedly detected under both planting densities, distributed across 14 chromosomes of soybeans. A hotspot region was identified on chromosome 19, from which seven candidate genes were detected. Based on haplotype and gene expression analyses, *Glyma.19g212800* (*SUS3*) and *Glyma.19g212700* (*GH9B13*) were found to be associated with significant phenotypic variations and were identified as candidate genes. RNA-seq analysis showed that DEGs were primarily enriched in the starch and sucrose metabolism pathways. The differential expression of *Glyma.19g212800* among soybean haplotypes was further validated by qRT-PCR. By participating in sucrose decomposition and polysaccharide metabolism processes, it regulated cellulose content, thereby affecting the soybean plant lodging. This study facilitated the dissection of genetic networks underlying lodging traits in soybean, which benefits the genetic improvement of high-yield soybean with dense planting.

## 1. Introduction

Soybean (*Glycine max* (L.) Merr.), as a major oil crop and a significant source of plant protein, is cultivated worldwide [1]. Recent advancements in breeding have emphasized the importance of high-density planting techniques for maximizing the yield potential of soybean cultivars [2,3]. The primary factors influencing soybean tolerance to high planting density include plant architecture and lodging resistance [4,5]. The morphological traits include agronomic characteristics such as plant height, stem diameter, number of branches, number of nodes on the main stem, and leaf-related traits [6,7]. These agronomic traits affect soybean tolerance to high-density, light interception rates, and cause competition for moisture, ultimately influencing grain yield.

Increasing planting density increased the risk of plant lodging, which led to significant reductions in crop yield and quality by reducing photosynthesis and nutrient transport. Since the Green Revolution, the development of cultivars with strong lodging resistance and optimal plant architecture has been consistently pursued by plant breeders. This effort laid the foundation for increasing crop yield and productivity through genetic improvement [7,8]. Lodging is a complex trait controlled by multiple QTLs and genes [9]. The study of quantitative trait loci associated with lodging and the relevant genes was of significant importance for guiding molecular marker-assisted selection (MAS) and for investigating the molecular basis of this trait.

Appropriately reducing plant height and increasing the main stem nodes were crucial for improving the lodging resistance of soybean. Plant height, however, is regulated by the flowering stage, with early flowering soybean varieties usually being shorter than late-flowering varieties [10,11,12], indicating a significant correlation between the degree of lodging and plant architecture. The stem strength [13], stem bending force, and root system morphology [14,15] are closely associated with lodging. In addition, multiple environmental factors (such as diseases, wind speed, and water stress) significantly influence the occurrence of soybean lodging [16,17]. Therefore, identifying the major quantitative trait loci (QTLs) and candidate genes associated with lodging resistance in soybeans was expected to contribute to improvement of the soybean lodging resistance. Multiple QTLs associated with lodging score have been deposited in SoyBase (http://www.soybase.org/). (accessed on 1 November 2024) Specht et al. found five QTLs on chromosomes 6, 12, and 19 using SSR and RFLP markers in F7:11 populations from the crossing of Minsoy × Noir1 [18]. In a separate study, Lee et al. identified four QTLs related to soybean lodging on chromosomes 4, 6, and 19 using 1536 SNP markers [19]. Most of the aforementioned QTLs were identified using low-density genetic linkage maps constructed from soybean RIL populations through SSR and RFLP markers. Owing to the limited size of natural populations, numerous QTLs exhibited wide confidence intervals, resulting in the mapping of only a limited number of loci under diverse environmental conditions. Therefore, the exploration of gene loci for tolerance to dense planting and cultivating high-yield and dense-tolerant varieties was beneficial for improving soybean yield. Genome-wide association study (GWAS) is a powerful tool for identifying links between phenotype variations and nucleotide polymorphisms in diverse natural populations [20]. It provides insights into the genetic basis of complex agronomic traits derived from natural allelic variation formed over multiple generations [21]. GWAS has been proven effective in pinpointing genes responsible for lodging resistance, with numerous examples reported in soybean. For instance, GWAS analysis conducted on 130 soybean lines identified two QTLs associated with stem lodging resistance, which were located on chromosomes 5 and 11 [14]. In a previous study, Tokachi nagaha was used as a parent and 137 varieties that were derived from it were subjected to association mapping, through which 27 significant SNPs were detected near major QTLs for agronomic traits [22]. Similarly, several examples were also reported in maize [23,24]. In *Brassica napus*, 92 SNPs and 50 SSR loci were identified by GWAS as being associated with lodging resistance and lignin-related traits [25]. Among 795 rice varieties, 33 pleiotropic loci were identified by GWAS, and *OsFBA2* was confirmed as a candidate gene conferring lodging resistance [26]. In wheat, GWAS has also been widely used to discover lodging resistance genes, with 14 significant SNPs and 13 QTLs identified in natural populations. Additionally, two candidate genes related to cellulose crystallinity, *TraesCS4B03G0029800* and *TraesCS5B03G1085500*, were identified [27]. Another GWAS employed a novel high-throughput phenotyping (HTP) method to assess the lodging trait in wheat, and identified a key genomic region on chromosome 2A [28].

The combination of favorable alleles at lodging-resistance loci was considered beneficial for achieving optimal plant architecture-suitable dense planting. In this study, whole-genome resequencing was performed on 338 soybean accessions to explore population structure and genetic diversity. Lodging scores were evaluated at planting densities of 300,000 and 150,000 plants/ha for the purpose of conducting a genome-wide association study. The results of the GWAS confirmed the presence of co-localized intervals, and was followed by screening using gene annotation and haplotype analysis. This study aims to provide new insights into the genetic mechanisms underlying lodging resistance in soybeans. The results are expected to facilitate the genetic improvement of soybean for tolerance to high planting density and resistance to lodging.

## 2. Results

### 2.1. Phenotype Variations of Lodging Traits in Soybean

**Figure 1:**
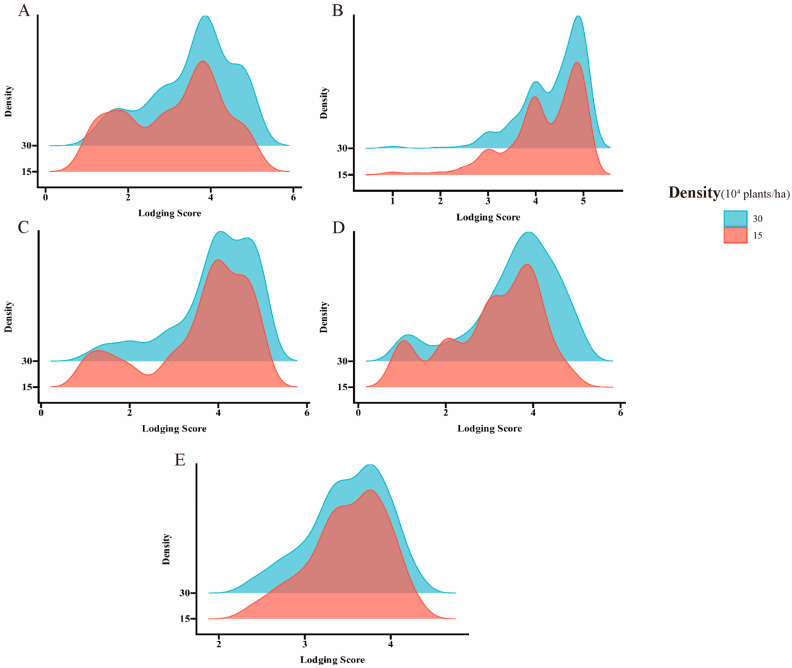
Frequency distribution histograms of lodging values and BLUP values under varying planting densities across different cultivation sites. (**A**) A density map of the 2022 SH site. (**B**) A density map of the 2023 SH site. (**C**) A density map of the 2023 DL site. (**D**) A density map of the 2023 HN site. (**E**) A density map of the BLUP value.

**Figure 2:**
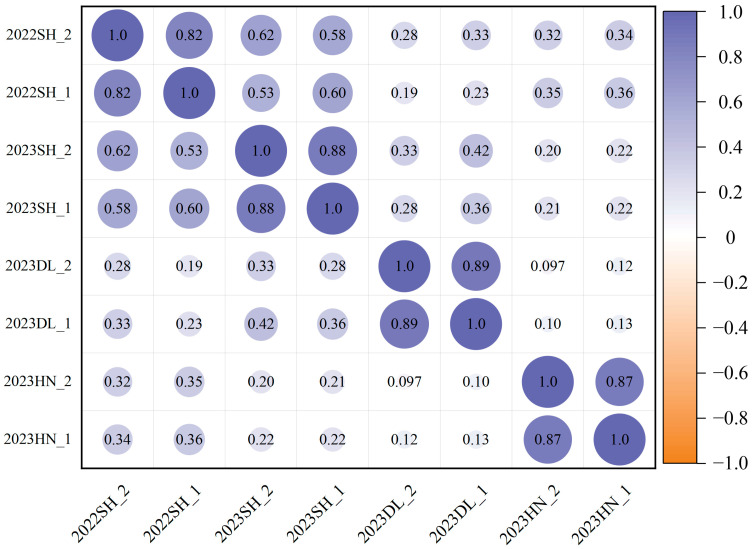
Pearson correlation analysis of 338 soybean germplasms for lodging at different planting densities.

Under the planting densities of 300,000 and 150,000 plants/ha, lodging exhibited approximately fivefold variation. The observed ranges were 1.05–5.00 and 1.00–5.00, with corresponding mean values of 3.85 ± 0.91 and 3.51 ± 1.04, respectively. The coefficient of variation spanned from 14.6% to 37.7%, and the broad-sense heritability under the two planting densities ranged from 0.52 to 0.55, indicating a broad range of variability (Table 1, Figure 1). The correlation analysis indicated that the significant correlation coefficients for the two-year repetitions at different densities in the Scientific Experimental Base of Shenyang Agricultural University are 0.62 and 0.58, respectively. Furthermore, a notable positive correlation was observed between different densities at the same experimental site, ranging from 0.82 to 0.89 (Figure 2).

### 2.2. Linkage Disequilibrium, Population Genetic Structure, and SNP Distribution

**Figure 3:**
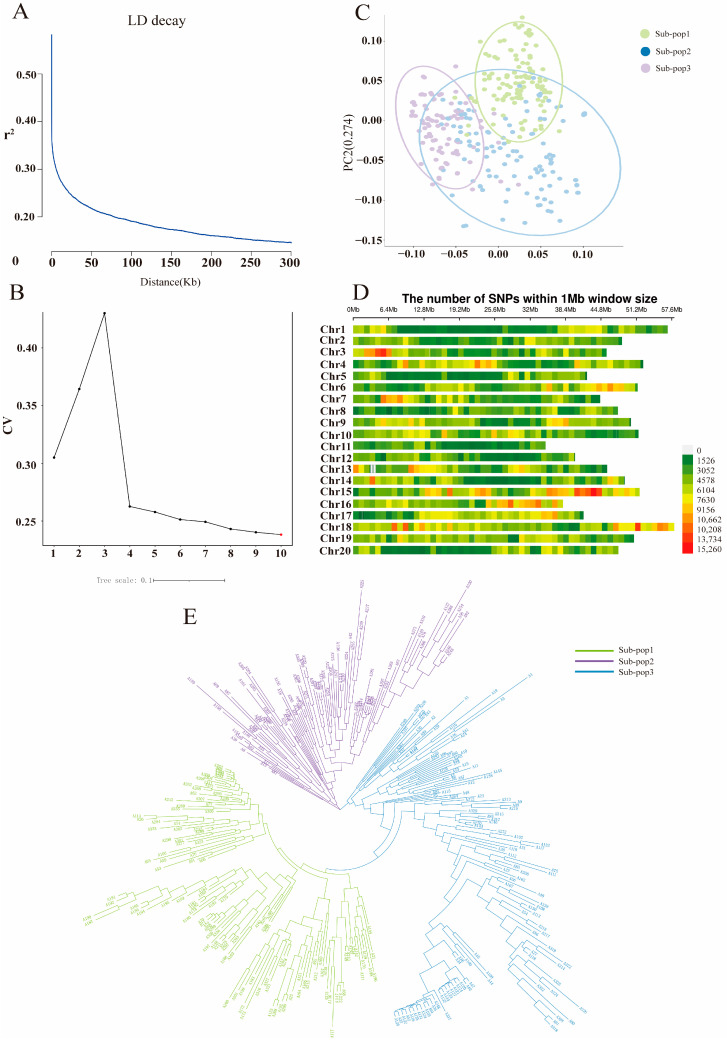
Analysis of population structure and linkage disequilibrium in 338 soybean samples. (**A**) LD analysis of the 338 germplasms. (**B**) Cross-validation error rate based on clustering of the 338 samples. (**C**) Principal component analysis of the 338 germplasms. (**D**) SNP diversity analysis across the 20 chromosomes of soybean. (**E**) Phylogenetic tree constructed using the neighbor-joining method for the 338 germplasms.

To further understand the genetic basis of trait variation, SNP diversity was analyzed in 338 soybean germplasm populations and their genomes were mapped using soybean Williams82 (Wm82.a2.v1) as a reference genome, which detected a total of 94,009,887 SNPs and 13,247,714 indels covering 20 chromosomes of the soybean genome (MAF > 0.05, missing genotype < 15%). Linkage disequilibrium (LD) decay was represented graphically, with the average r^2^ value across the entire genome being 0.19. The LD decay initiated at 0.61 and reached half-decay at r^2^ = 0.31. When the SNP distance reached approximately 100 kb, the coefficient of determination (r^2^) decreased to half of its maximum value (Figure 3A). The optimal K-Means clustering value was observed at K = 3, confirming the classification of 338 soybean genotypes into three subgroups (Figure 3B and Figure S1). Principal component analysis (PCA) indicated that the accessions were categorized into three distinct subgroups (Figure 3C), which was consistent with the phylogenetic tree. All 20 chromosomes were covered by the detected SNPs, with the highest and lowest SNP numbers observed on Chr.13 (218,699) and Chr.18 (53,502), respectively (Figure 3D). Chr.11 and Chr.16 showed the lowest and highest SNP densities of 1538.74 SNPs/Mb and 49.02 SNPs/Mb, respectively. The heat map and dendrogram of the kinship matrix were generated based on the polymorphic SNPs, which revealed three distinct clusters of the soybean accessions (Figure 3E).Therefore, the genome-wide LD pattern was examined to estimate the number of haplotypes containing SNPs for subsequent candidate gene interval identification.

### 2.3. Genome-Wide Association Analysis

**Figure 4:**
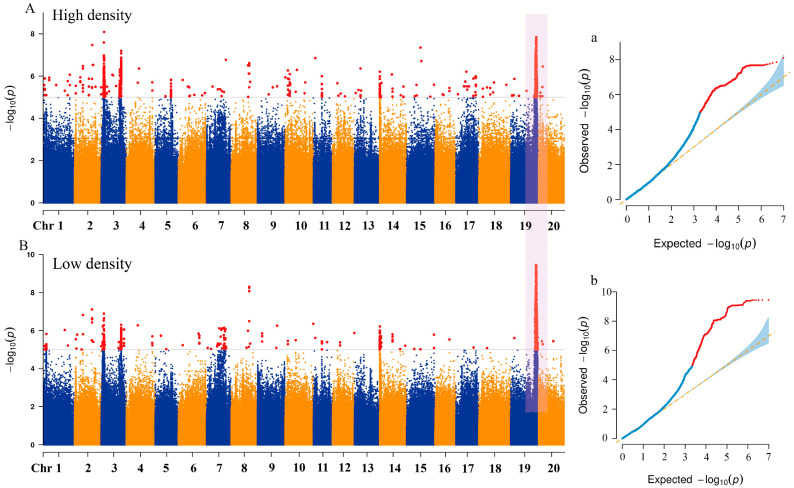
Genome-wide association analysis using Manhattan and QQ plots of 338 germplasms for lodging traits at different planting densities based on BLUP values at four locations. (**A**) Manhattan plot association results at 300,000 plants/ha. (**B**) Manhattan plot association results at 150,000 plants/ha (Red dots represent SNPs exceeding the genome-wide significance threshold, and the purple shaded region indicates a co-localized hotspot identified under both planting densities). (**a**) QQ plot at 300,000 plants/ha. (**b**) QQ at 150,000 plants/ha plot (The blue curve represents the observed –log_10_(p) distribution under the null hypothesis. The red curve indicates deviations beyond the genome-wide significance threshold. The blue shaded area represents the 95% confidence interval under the null hypothesis).

To identify the genetic basis of natural variation in the lodging resistance of soybeans under different density conditions, a total of 9,400,987 high-quality SNP markers were used, and the phenotypic BLUP values of the lodgings score in four environments in 2022 SH, 2023 SH, 2023 HN, and 2023 DL were applied to conduct GWAS analysis on 338 soybean accessions using the mixed linear model MLM (Q + K) model (Figure 4). Under high-density conditions, a total of 135 SNP loci were significantly associated with lodging score (−log10 (*p*) ≥ 5), and a total of 102 SNP loci were significantly associated with lodging score (−log10 (*p*) ≥ 5) in low-density conditions, with association signals detected across all 20 chromosomes of soybean (Table S1). Twenty significant SNPs were consistently identified under both planting density conditions, indicating that these SNPs are stable markers for comparison. The associated SNP loci were distributed across 14 chromosomes (Table 2). Significant SNP loci were detected on chromosomes 4, 7, 9, 10, 11, 15, 18, and 20 (one significant SNP per chromosome), whereas two significant SNPs were identified on chromosomes 1, 2, 3, 8, 14, and 19. Additionally, a hotspot region was identified on chromosome 19, with the SNP peak position locus located at 46,692,661 bp. The −log10 (*p*) values under different planting densities were 7.85 and 9.45, explaining the highest phenotypic variation at 9.16 and 10.99%, respectively. The results suggested that multiple genes associated with SNPs located on different chromosomes collectively influence the lodging resistance of soybeans. The repeatedly detected linked gene loci under different environments provided a solid foundation for dissecting the genetic network of soybean lodging resistance.

### 2.4. Screening and Identification of Candidate Genes

In order to identify candidate genes related to lodging resistance, candidate genes were mined within a 100 kb interval both upstream and downstream of the significant SNP sites found from the GWAS co-localization results under the conditions of 300,000 plants/ha and 150,000 plants/ha. Based on LD decay analysis, a total of 47 genes were detected within the 100 kb regions flanking 13 SNPs, all of which contained non-synonymous mutations in their coding regions (Table S2). Since non-synonymous mutations in the coding region caused changes in amino acids substitutions or the premature termination of translation, thereby altering plant phenotypes, these genes were defined as pre-candidate genes. A functionally annotated gene was screened at 2,931,250 bp on chromosome 1, encoding a cysteine-rich RLK (RECEPTOR-like protein kinase). At 5,536,931 bp on chromosome 3, four functionally annotated genes were identified, encoding LRR and NB-ARC domains that contain a disease-resistance protein, RNI-like superfamily protein, a homolog of yeast autophagy 18 (ATG18) G, and protein kinase family protein, respectively. A total of 42 genes were identified from the genetic region surrounding Chr19:46692661. Previous studies demonstrated that the regulation of soybean meristematic tissue, plant cell wall synthesis, the regulation of sucrose, cellulose, lignin synthesis and metabolism, as well as hormone signaling-related genes, modulated the lodging resistance of soybean [29,30]. Therefore, functional annotation results of NCBI and SoyBase databases were used as references, and the biological pathways of each gene were screened and predicted by analyzing the homologous genes of *Arabidopsis thaliana*. Based on the above conditions, a total of seven candidate genes were identified, all located on chromosome 19 (Table 3). Among them, *Glyma.19G200800* (*NF*-*YA10*) was annotated as an encoding enrichment of nuclear factor Y subunit A10. *Glyma.19G203400* (*AMP1*) was predicted to encode glutamate carboxypeptidase, involved in abscisic acid, oxidation, and abiotic stress responses, and carbon and amino acid metabolism. *Glyma.19G212700* (*GH9B13*) was predicted to encode a glycosyl hydrolase involved in the metabolism of carbohydrates, including cellulose catabolism. *Glyma.19G216600* (*SWN*) SET domain-containing protein, encoding a polycomb group protein, was also identified. *Glyma.19G215500* (*BXL2*) was predicted to encode a protein similar to a beta-xylosidase located in the extracellular matrix. *Glyma.19G221700* (*WRKY35*) was predicted to encode a WRKY family transcription factor that binds to DNA with a specific nucleotide composition. Therefore, seven genes were identified putative candidate genes.

### 2.5. Haplotype Analysis of Candidate Genes Glyma.19G212800 and Glyma.19G212700

**Figure 5:**
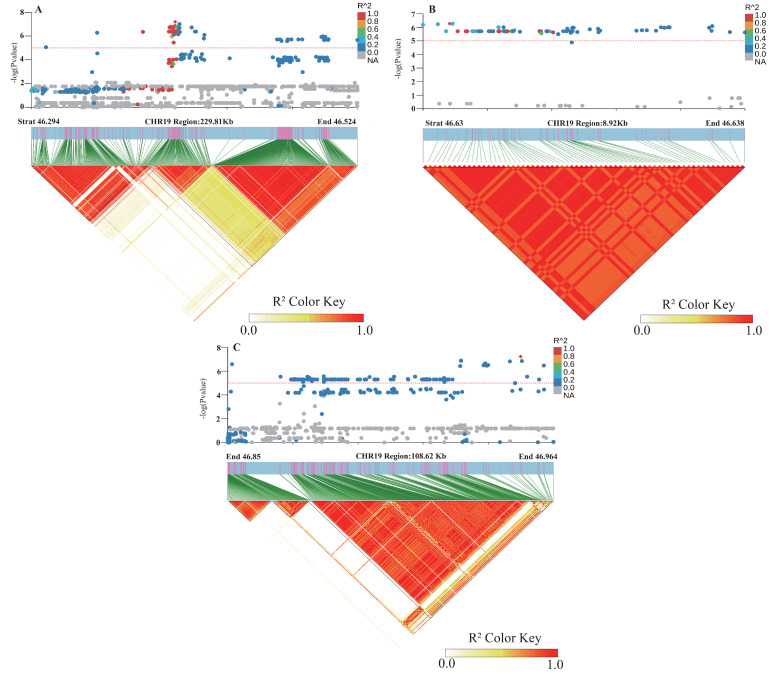
Linkage disequilibrium map (LD) of SNP Chr19.:46692661 on chromosome 19 for soybean lodging resistance.

**Figure 6:**
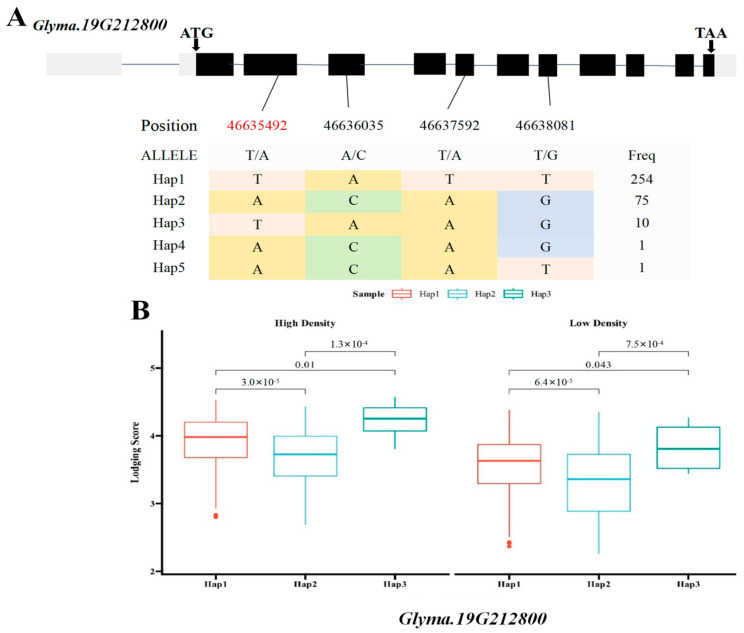
Box plots of the gene structure, haplotypes, and phenotypic variation of each haplotype of *Glyma.19G212800*. (**A**) Gene structure of *Glyma.19G212800*; gray blocks represent UTR regions and black blocks represent exons. The table shows the haplotypes of the gene. (**B**) Box plots of the phenotypic variation of each haplotype. The figure shows the lodging phenotype under high-density and low-density planting conditions. The *p* values of each of the three haplotypes calculated by using ANOVA are marked in the figure. (SNPs shown in red font indicate non-synonymous mutations).

**Figure 7:**
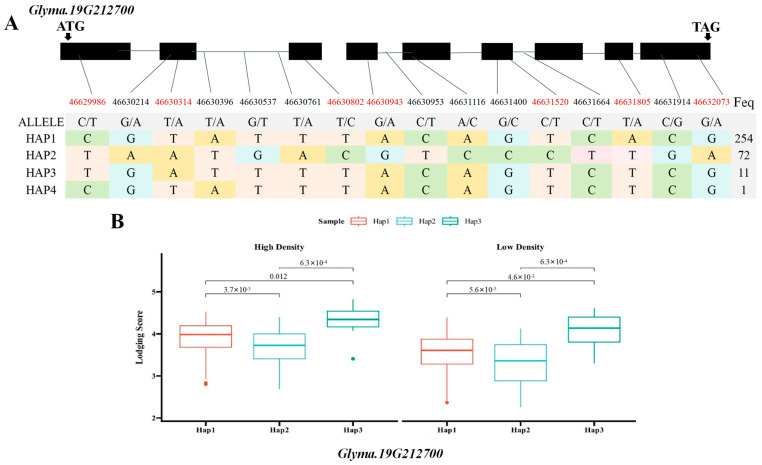
Box plots of the gene structure, haplotypes, and phenotypic variation of each haplotype of *Glyma.19G212700*. (**A**) Gene structure of *Glyma.19G212700*; gray blocks represent UTR regions and black blocks represent exons. The table shows the haplotypes of the gene. (**B**) Box plots of the phenotypic variation of each haplotype. The figure shows the lodging phenotype under high-density and low-density planting conditions. The *p* values of each of the three haplotypes calculated using ANOVA are marked in the figure(SNPs shown in red font indicate non-synonymous mutations).

Based on the previous literature and the soybean expression database, SNPs within two of the seven major candidate genes, the *Glyma.19G212800* gene encoding the sucrose synthase activity (SUS) protein and the *Glyma.19G212700* gene, involved in the polysaccharide decomposition process, were simultaneously detected in the BLUP value GWAS results under the planting densities of 300,000 and 150,000 plants/ha. This locus was detected at high frequency across various environments, indicating stable inheritance and significant contribution. Therefore, the genomic region near Chr19.:46692661 was extracted for local Manhattan plot and LD Block analysis. The local haplotype blocks formed by SNPs were abundant, highly interlinked to each other, and exhibited a high degree of disequilibrium (Figure 5A–C). The haplotype analysis of e *Glyma.19G212800* revealed the presence of four major single-nucleotide variants with significant differences. Among the six identified haplotypes, the statistical analysis of haplotype frequencies revealed 338 accessions and ultimately revealing three major haplotypes (Hap1, Hap2, and Hap3) in *Glyma.19G21280*, collectively representing over 99% of the population. Hap1 was the most prevalent, comprising 254 lines, followed by Hap2 (75 lines) and Hap3 (9 lines), respectively. These three major haplotypes differed at four coding region SNPs: T/A variation at 46,635,492 bp, A/C variation at 46,636,035 bp, T/A variation at 46,637,592 bp, and T/G variation at 46638081 bp (Figure 6A). Statistical tests revealed significant differences in lodging score traits corresponding to three main haplotypes (Hap1, Hap2, and Hap3) at different planting densities (*p* = 3.0 × 10^−5^, *p* = 0.01, *p* = 1.3 × 10^−4^, *p* = 6.4 × 10^−5^, *p* = 0.043, *p* = 7.5 × 10^−4^), with Hap2 exhibiting the lowest average score. At a planting density of 300,000 plants/ha, the lodging score of Hap1 increased by 0.24 compared with that of Hap2, and at a planting density of 150,000 plants/ha, it was 0.28 higher (Figure 6B). Consequently, Hap2 emerged as a distinct haplotype under different planting densities. Haplotype analysis of *Glyma.19G212700* identified four haplotypes based on 14 coding region SNPs, including seven non-synonymous mutations (Figure 7A). Hap1, Hap2, and Hap3 were present in 254, 72, and 11 lines, respectively, accounting for over 99% of the total accessions. An ANOVA test indicated that under planting densities of 300,000 and 150,000 plants/ha, the lodging score of varieties containing Hap3 was significantly higher than that of varieties containing Hap2 and Hap1 (*p* = 3.7 × 10^−5^, *p* = 0.012, *p* = 6.3 × 10^−4^, *p* = 5.6 ×10^−5^, *p* =4.6 × 10^−3^, *p* = 1.6 × 10^−4^), with the lowest lodging score observed in Hap2. Therefore, Hap2 was inferred to be a favorable haplotype (Figure 7B). In recent years, breeders have increasingly prioritized soybean varieties with resistance to lodging and tolerance to high planting densities. Thus, *Glyma.19G212800* and its Hap2 haplotype were considered key contributors to soybean lodging resistance. Based on these findings, *Glyma.19G212700* and *Glyma.19G212800* were identified as major candidate genes within the QTL on chromosome 19 associated with lodging resistance in soybean.

### 2.6. Transcriptome Analysis and qRT-PCR Validation

**Figure 8:**
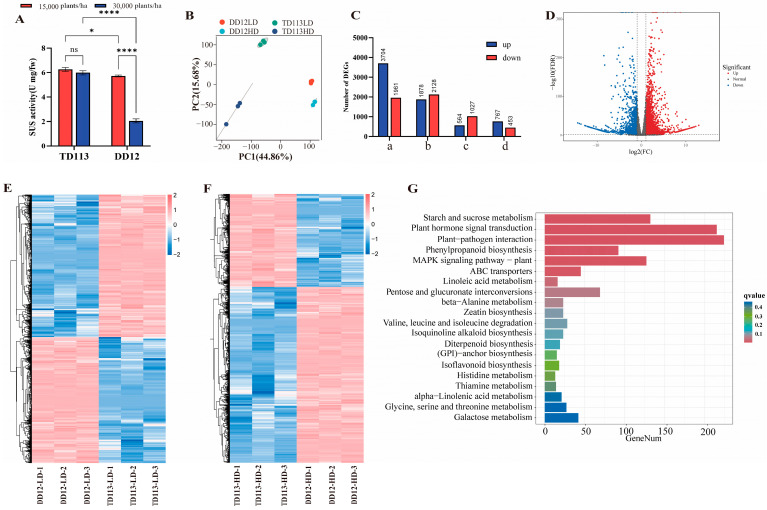
Transcriptome analysis of stem internodes 1–5 of soybean varieties TD113 and DD12 under different planting densities. (**A**) Changes in sucrose synthase activity under different planting densities. (**B**) Principal component analysis of transcriptome changes in TD113 and DD12 under different planting densities. (**C**) Differences in the number of upregulated and downregulated differentially expressed genes (DEGs) among different groups. (**D**) Volcano plot of DEGs in TD113 and DD12 under high planting density. (**E**) Heatmap of DEGs in TD113 and DD12 under low planting density. (**F**) Heatmap of DEGs in TD113 and DD12 under high planting density. (**G**) KEGG enrichment of DEGs with q-value (ns, not significant; *, *p* < 0.05; ****, *p* < 0.00001).

To further validate the candidate genes, two soybean varieties exhibiting extreme haplotypes were selected: the lodging-resistant variety Tie Dou 113 (TD113) and the lodging-susceptible variety Dan Dou 12 (DD12). The activity of sucrose synthase (SUS) was measured. As shown in Figure 8A, the SUS enzyme activity in the stem internodes of TD113 remained relatively stable under high-density planting (300,000 plants/ha) compared to the low-density planting (150,000 plants/ha), whereas in DD12, the stem internodes increased significantly. Following the increase in planting density, the SUS in the stem of DD12 showed a pronounced decrease and was significantly lower than that of TD113 under the same conditions. These results suggested that TD113 maintained a relatively high level of SUS in the stem under high-density planting conditions, facilitating sucrose accumulation. Transcriptomic changes in stem tissue under high-density planting were assessed using RNA sequencing. Firstly, principal component analysis (PCA) revealed the separation of the PC1 and PC2 factors of samples from different varieties, accounting for 44.86% and 15.68% of the total transcriptome variation, respectively (Figure 8B). Under high-density planting conditions, 564 genes were significantly upregulated and 1027 genes were significantly downregulated, while in DD12, 767 genes were significantly upregulated and 453 genes were significantly downregulated, respectively. Compared with TD113, 3704 genes were upregulated and 1961 downregulated in DD12 compared with TD113 under high-density planting, while under normal planting density conditions, 1878 genes were upregulated and 2128 genes were downregulated (Figure 8C–F). KEGG enrichment analysis indicated that these differentially expressed genes (DEGs) were primarily involved in starch and sucrose metabolism, plant–pathogen interaction, plant hormone signal transduction, and phenylpropanoid biosynthesis (Figure 8G).

**Figure 9:**
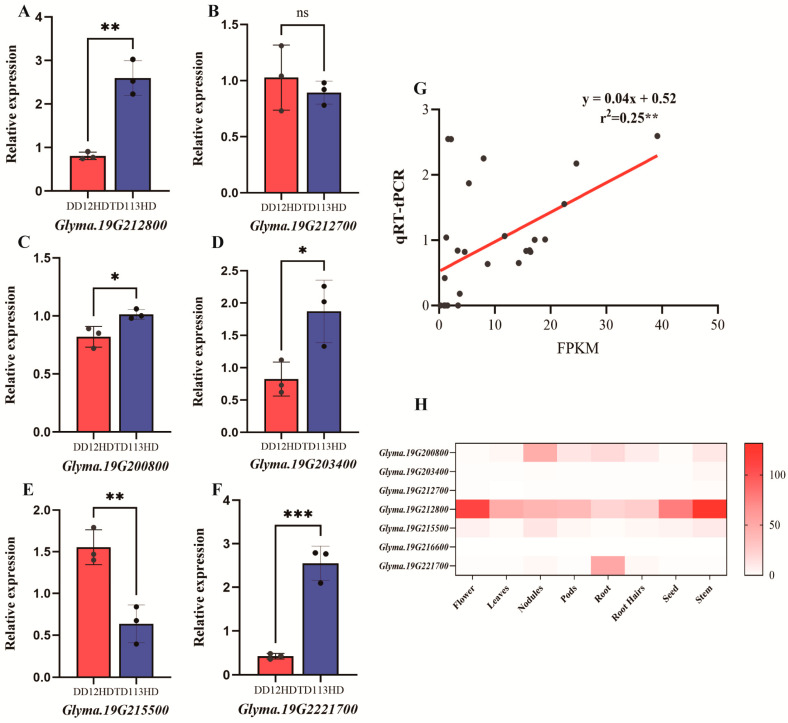
(**A**–**F**) qRT-PCR validation of candidate genes. (**G**) Correlation analysis in transcript changes of 6 DEGs in RNA-seq (*x* axis) and qRT-PCR experiment (*y* axis). Gene relative expression was calculated by 2^−ΔΔCt^. (**H**) Heatmap of candidate gene expression levels in different soybean tissues. Statistical analysis was performed using Student’s *t*-test (ns, not significant; *, *p* < 0.05; **, *p* < 0.01, ***, *p* < 0.0001).

To verify the accuracy of transcriptome sequencing, changes in the expression levels of candidate genes under high planting density were analyzed in contrasting varieties using qRT-PCR (Figure 9). In the lodging-resistant variety TD113, the expression levels of *Glyma.19G212800*, *Glyma.19G200800*, *Glyma.19G203400*, and *Glyma.19G221700* were significantly upregulated, while those of *Glyma.19G212700* and *Glyma.19G215500* were relatively low. The expression of *Glyma.19G22160* was not detected (Figure 9A–F). Moreover, qRT-PCR results were significantly positively correlated with RNA-seq (*p* = 0.006) (Figure 9G). According to public tissue expression data, *Glyma.19G212800* exhibited relatively high specificity in expression during the reproductive growth stages of soybeans, particularly in flowers, stems, and seeds. These findings suggested that *Glyma.19G212800* played a specific role in stem development and growth in soybeans (Figure 9H).

## 3. Discussion

### 3.1. Analysis of Genetic Variation for Lodging Score Traits

Lodging traits were affected by genetic background, environmental factors, and cultivation conditions [31]. Improving the lodging resistance was considered an effective approach to increase yield under the conditions of increased planting density. Since the Green Revolution, breeding soybean varieties with stronger stems and shorter internodes has become a direction for improving soybean lodging resistance [32]. Currently, the application of GWAS has been validated as an effective method to identify traits associated with lodging resistance in soybeans, with multiple studies successfully identifying several QTLs [19,33,34]. The lodging score was used to evaluate the degree of stem inclination in field-grown soybean plants, enabling comprehensive assessment based on actual growth conditions. Lodging classification in soybean varieties, both domestically and internationally, was primarily based on direct field evaluation [35,36,37,38,39,40]. BLUP values of lodging scores under two planting densities showed substantial variation and followed a normal distribution, with the coefficient of variation ranging from 14.6% to 37.7%. In the soybean recombinant inbred line population, the range of lodging phenotypic variation over three consecutive years was 37–40% [41]. These findings indicated that lodging exhibited greater potential for genetic improvement and was strongly influenced by environmental factors. GWAS enabled the analysis of genotype and phenotype associations, facilitating the identification of key loci and favorable haplotypes associated with important traits [42]. In this study, a total of 9,400,987 SNPs were detected for population structure analysis, and 338 soybean accessions were classified into three subpopulations, indicating considerable genetic diversity within the population [43]. The evaluation of lodging in different environments was essential for studying genetic variation in lodging. In this study, based on 338 germplasms as an associated population, we elucidated the detailed characterization of lodging-associated genes by using GWAS; GWAS was performed to identify lodging-related genes by integrating phenotypic and SNP data from four environments in 2022–2023, in combination with haplotype and candidate gene analysis.

### 3.2. Comparative Analysis of Loci Associated with Lodging Resistance

QTLs that were consistently detected across multiple genetic backgrounds and environments were considered to be plausible. Previous studies identified numerous markers and genomic regions associated with lodging traits through QTL and GWAS analysis [22]. Many QTLs had broad confidence intervals, with only a few loci that were consistently mapped across different environments. Additionally, many lodging-related QTLs in soybeans were detected within recombinant inbred line (RIL) populations, but were less prevalent in natural populations [44]. In this study, 338 soybean accessions were analyzed using the MLM model and Q + K matrix, and GWAS identified 20 SNPs significantly associated with lodging scores. Compared with previous studies, we found that 11 out of 20 SNPs identified by the genome-wide association study (GWAS) were located within previously reported quantitative trait loci (QTL) regions. Among them, SNPs located on chromosomes 2, 7, 8, 9, 10, 11, 15, 18, and 20 were newly identified loci associated with lodging resistance [18,19,20,22,37,38,39,45] (Figure 4, Table 2). The SNP Chr01:39325847 located on chromosome 1 was located within the QTL interval reported by Kim et al. [46] (Gm01:6926577–Gm01:49435179). The significant SNP Chr02:260473219 on chromosome 2 was located within the QTL interval reported by Orf et al. [42]. The SNPs Chr03:35831309 and Chr03:36451125 were located 132.7 and 194.6 kb from the previously reported QTL Satt237 [43]. SNP Chr19:44477717 was included in a major QTL region previously reported by Mansur and Lee et al. [44,45]. The SNP located at Chr19:46692661 overlapped with the QTL mapped by Lee et al. (Gm19:45182458–Gm19:48028990). This SNP explained 9.16–10.99% of the phenotypic variation, indicating a significant association between this hotspot region and the lodging trait. Lodging is a complex trait regulated by multiple genes. The lodging trait is significantly correlated with plant height, maturity, stem diameter, and yield-related traits [46,47]. These findings demonstrated the genetic diversity of the accessions and confirmed the reliability of the results. The results indicated the diversity of the materials used and the reliability of the research. The identified genetic loci had a high level of credibility, and these loci contained important genes that were stably regulated in different environments to control soybean lodging and related traits. This region represented a key target for future studies on lodging resistance and provided a foundation for marker-assisted selection in soybean breeding.

### 3.3. Analysis of Candidate Genes for Lodging Resistance

Lodging was reported to be associated with plant hormone synthesis and transduction, cell wall composition, sucrose, lignin, and cellulose synthesis [48]. A total of 47 non-synonymous SNPs were identified within the LD decay interval based on significant SNP loci. Among them, seven candidate genes associated with lodging traits were identified at the SNP-Chr19.:46692661 locus by integrating gene function annotation and homologous gene function in *Arabidopsis* (Table 3 and Table S2). By altering the structure of the cell wall in wheat through hormones and ROS pathways, cell wall loosening occurs, restricting cell elongation, and enhancing lodging resistance [49]. *Glyma.19G203400* encodes glutamate carboxypeptidase, an integral membrane protein that is associated with endoplasmic reticulum where it co-localizes with *AGO1*. Various alleles exhibited increased cotyledon number and leaf initiation rates, altered flowering time and photomorphogenesis and increasing the level of cytokinin biosynthesis. It is also involved in ethylene-enhanced hypocotyl elongation under light conditions [50]. In maize, *ZmAMP1* regulated yield-related traits by regulating *GRMZM2G050137* or *GRMZM2G117961*. The varieties into which its allele was introduced had short and strong stems, which improved the lodging resistance and density tolerance of maize [13,51]. *Glyma.19G200800* encodes nuclear factor Y, subunit A10 (*NF-YA10*) CCAAT-binding transcription factors, which regulate transcription from DNA templates. *NF-YA10* was expressed in the SAM and leaf vascular system. *YUC2*, a crucial gene regulating auxin homeostasis, was directly targeted by *NF-YA2* and *NF-YA10*. Increasing the expression of *NF-YA2* and *NF-YA10* suppressed *YUC2* activity, thereby reducing endogenous IAA levels. A reduction in IAA content led to the downregulation of *PIN* and *ARFs* family expression. Transcriptional repressors *ARF 1* and *ARF 2* directly bound to promoters containing auxin response elements (TGTCTC), inhibiting IAA signaling and other transcriptional targets, and thus affecting soybean plant development and lodging resistance [52,53,54,55]. The *Glyma.19G215500* gene is a member of the GH3 family and encoded the BXL protein. BXL is an enzyme that degraded cellobiose and oligosaccharides into xylose monomers, serving as a rate-limiting enzyme in the degradation of cell wall hemicellulose [56]. The *AtBXL2* gene was highly expressed in the nodes and internodes of *Arabidopsis thaliana*. In poplar, this gene affects the secondary cell walls and hemicellulose, suggesting that it regulates hemicellulose metabolism and cell wall plasticity in soybean stems, thereby affecting their lodging resistance [57]. The candidate gene *Glyma.19G216600* (*SWN*), located on chromosome 19, encoded a polycomb group protein that synthesized protein complexes such as *VRN2*(VERNALIZATION 2), *VIN3* (VERNALIZATION INSENSITIVE 3), and *CLF* (CURLY LEAF), and regulated secondary cell wall biosynthesis [58]. The rice homolog *OsSWN1* (*Oryza sativa* Secondary Wall NAC 1) is a key regulatory factor of secondary cell wall formation capable of inducing its development [59]. Cloned *SWNs* genes in bamboo also function as strong regulators of secondary cell wall formation, driven by activation from the C-terminal domain, and serve as genetic tools for secondary cell wall engineering [60]. Therefore, it was speculated that this gene improved soybean stem strength and biomass, thereby improving soybean lodging resistance. *Glyma.19G221700* encodes a WRKY family transcription factor that binds to DNA with sequence-specific motifs. In *Populus tomentosa*, *WRKY35* directly regulates the expression of monolignin biosynthesis genes such as *MgPAL1* and *MgCOMT* by binding to their promoters [61]. Therefore, it was inferred that this gene affects lignin monomer synthesis and impacts the lodging resistance of soybean stems.

### 3.4. Haplotype Analysis, Transcriptome Profiling, and qRT-PCR Validation of Candidate Genes

Haplotypes refer to combinations of multiple markers on a single chromosome, currently widely used in crops such as rice, soybeans, maize, and others [62]. Previous studies suggested that enhancing soybean lodging resistance was beneficial for establishing a reasonable plant type, improving photosynthetic efficiency, and increasing yield by optimizing planting density. In this study, *Glyma.19g212800* (*SUS3*) and *Glyma.19g212700* (*GH9B13*) were identified as candidate genes by integrating evidence from expression profile databases, literature review, and haplotype analysis. The protein encoded by *Glyma.19g212700* was a glycosyl hydrolase enzyme involved polysaccharide metabolism. It was homologous to *AtGH9B13* from the *GH9B* gene family, participating in the chemical reactions and pathways of cellulose decomposition. The cellulase encoded by this gene was mainly involved in the repair or arrangement of cellulose microfibrils during cellulose biosynthesis in plants. Recent studies revealed that *OsGHB 1*, *OsGHB 3*, and *OsGHB 16* could reduce the enzymatic activity related to cellulose crystallinity in rice. Although limited studies were available on this gene, *Glyma.19g212700* was hypothesized to regulate enzymatic activity linked to cellulose crystallinity in plants, thereby influencing cellulose synthesis in stems soybean stems [63]. The protein encoded by *Glyma.19g212800* was sucrose synthase (SUS), which participated in the sucrose synthesis metabolism process. Sucrose synthase (SUS) catalyzed the reversible conversion of sucrose and UDP into UDPG and fructose. Cellulose is the primary component of the cell wall, and cellulose synthase (CESA) uses UDP-glucose (UDPG) as a substrate to catalyze cellulose biosynthesis. SUS in the fiber of cotton provided a UDPG substrate to regulate cellulose biosynthesis [64]. However, currently, there were few reports on the regulation of plant lodging with this gene. A mechanism model of the *SUS3* gene had been proposed in rice, which decreased cellulose crystallinity by positively regulating cellulose and hemicellulose biosynthesis. This resulted in the thickening of the plant cell wall, significantly increasing the lodging resistance of transgenic rice [65]. The variant Chr19_46635492 was located in the exonic region of the candidate gene, leading to a non-synonymous mutation. Through haplotype analysis, Hap2 was identified to confer beneficial lodging resistance, with a frequency of 22.19% among 338 accessions. Therefore, Hap2 represented the main favorable haplotype in *Glyma.19g212800* (Figure 6). These findings indicated that multiple SNP sites of candidate genes underwent non-synonymous mutations, and Hap2 was identified as a favorable haplotype associated with lodging resistance. Among 338 germplasms, the frequency of Hap2 occurrence was 21.30%, suggesting a positive selection of these breeding choices in this study. The RNA-seq results identified 5565 differentially DEGs, which were mainly enriched in the pathways of starch and sucrose metabolism, plant–pathogen interaction, plant hormone signal transduction, and phenyl propanoid biosynthesis pathways. The *Glyma.19g212800* gene was enriched in the starch sucrose metabolism pathway and was significantly downregulated. This result was also verified by qRT-PCR. However, *Glyma.19g212700* was not significantly expressed. Therefore, *Glyma.19g212800* may regulate soybean lodging by affecting starch and sucrose metabolism in soybean stems. Therefore, HAP2 represented a superior haplotype. Its SNP variant was considered a functional polymorphism and was used to develop molecular markers for assisting the selection of soybean lodging resistance traits. The utilization of these superior haplotypes also promoted the breeding of soybean varieties with enhanced lodging resistance.

Incorporating favorable alleles at lodging-resistance loci into soybean breeding programs was considered beneficial for achieving the ideal plant architecture for dense planting. Whole-genome resequencing was performed on 338 soybean accessions to explore population structure and genetic diversity. The lodging score was set at the densities of 300,000 and 150,000 plants/ha for the purpose of conducting a genome-wide association study (GWAS). The GWAS results confirmed the co-localized intervals, followed by screening using gene annotation and haplotype analysis. This study aims to provide new insights into the genetic mechanisms underlying lodging resistance in soybeans. The findings supported genetic improvement for enhanced lodging resistance and adaptation to high planting density.

## 4. Materials and Methods

### 4.1. Plant Materials, Field Experiments and the Measurement of Trait

Soybean is a short-day crop and the photoperiod plays a critical role in its growth, development, and reproductive phases. To minimize the adverse effects of germplasm introduction, it is essential to avoid the effect of lodging caused by a south cultivar introducing northward, or a north cultivar introducing southward. This study evaluated 338 soybean germplasms, including local lines developed over different decades in Liaoning Province and introduced lines from Japan and the United States at similar latitudes, forming the natural population used for analysis (Table S3). All plant materials were planted at the Experimental Base of Shenyang Agricultural University in 2022 and 2023 at three locations in Liaoning Province: Shenyang (2022SH and 2023SH, 41.82° N, 123.57° E), Hunnan District (2023HN, 41.75° N, 123.69° E), and Pulandian District, Dalian (2023DL, 39.54° N, 122.20° E). The experiment was a completely randomized block design, with 5 rows per entry, a row length of 5 m, a row spacing of 0.6 m, and a plant spacing of 5 cm and 11 cm for the respective planting densities of 300,000 and 150,000 plants/ha. Plant thinning was performed 20 days after sowing to ensure proper density, with management practices aligned with conventional field management.

The lodging score of each plot was measured before harvest at maturity (R8). The specific criteria are as follows, with the traits measured using a 1 to 5 scale: 1 = Almost all plants erect, 2 = All plants leaning slightly or a few plants down, 3 = All plants leaning moderately (45 degrees), or 25–50% of plants down, 4 = All plants leaning considerably, or 50–80% of plants down, 5 = Prostrate, almost all plants down [18,19].

### 4.2. Genotyping and SNP Calling

Fresh leaf samples were collected from 338 soybean accessions cultivated at the V2 growth stage and subsequently placed in liquid nitrogen, with final storage at −80 °C. The DNA extraction from the young leaf of soybean accession was performed using the CTAB method. Library construction and sequencing used BGIT7 sequencing platforms (MGI Tech Co., Ltd., Shenzhen, China). The library was constructed utilizing the MGIEasy Universal DNA Library Prep Kit V1.0 (Product No. 100 000 5250, MGI) [66,67]. Following this, the genomic DNA was fragmented, and the selected fragments underwent end repair and 3′-end adenylation before the ligation of adapters. Subsequently, the circularization reaction system and program were established, resulting in single-stranded cyclized products, while uncyclized linear DNA molecules were digested. Rolling cycle amplification was employed to replicate the single-stranded circular DNA molecules, leading to the formation of DNA nanoballs (DNBs) that contained multiple copies of DNA. High-quality DNBs were then loaded onto patterned nanoarrays using the high-intensity DNA nanochip technique and sequenced through combinatorial Probe-Anchor Synthesis (cPAS). Raw sequence reads were filtered by discarding those with a N content > 10% or a base quality score (Q score) < 10. The raw sequencing reads were aligned to the soybean reference genome Wm82.a2.v1 (https://phytozome.jgi.doe.gov, accessed on 1 November 2024) using the Burrows-Wheeler Aligner (BWA, v0.7.17), generating SAM files that were subsequently converted into BAM format for downstream variant analysis. The aligned BAM files were processed using BCF tools (v1.12) to sort the alignments, remove PCR duplicates, and perform variant calling. Only biallelic sites with a missing rate ≤ 15% and a minor allele frequency (MAF) > 0.05 were retained. Genotype imputation was then performed using Beagle (v5.1). After quality filtering and genotype imputation, a total of 4,432,394 high-quality SNPs were retained for downstream analysis.

### 4.3. Linkage Disequilibrium, Population Genetic Structure, and SNP Distribution

The first 20 principal components of the population were obtained using PLINK, and their significance was tested using EIGENSOFT v8.0.0. We selected the top k principal components with a significance level of *p* < 0.05 to serve as covariates for subsequent analyses. For individual *i*, the SNP at position *k* is represented by [0, 1, 2]: a value of 0 indicates that individual *i* is homozygous for the reference allele; a value of 1 indicates heterozygosity; and a value of 2 indicates that individual *i* is homozygous for the non-reference allele. M is a matrix of standard genotypes with dimensions *n* × S, and Formula (1) is as follows:

(1) $${d_{1k}}^{\prime }=\frac{d-E(d_{k})}{\sqrt{E(d_{k})\times (1-E(d_{k})/2)/2}},$$

*E*(*d_k_*) denotes the mean value of d_k_, and the individual sample covariance *n* × *n* matrix is calculated via X = MMT/S. The average LD decay was plotted through the R language. The LD decay rate of the population was measured as the chromosomal distance, where the mean r2 decreased to half of its maximum value [68]. Phenotypic variation explained (PVE) was calculated according to Formula (2):

(2) $$SNP(PVE)=\frac{\left[2*\left({beta}^{2}\right)*\left(1-MAF\right)\right]}{\left[2*\left({beta}^{2}\right)*MAF\left(1-MAF\right)+{\left(se\left(beta\right)\right)}^{2})*2*N*MAF*(1-MAF)\right]}$$

The symbols are defined as follows: beta: effect value in GWAS; MAF: minor allele frequency of SNP; se(beta): standard error (se) of effect value in GWAS; N: number of individuals involved in the analysis of this SNP in GWAS.

Population structure was inferred using the software ADMIXTURE v1.3.0. The distribution of genetic components was examined under different values of K (where K represents the assumed number of ancestral populations). Finally, the optimal number of ancestral groups was determined by identifying the K value at which the cross-validation (CV) error reached its minimum.

### 4.4. Genome-Wide Association Study

Genome-wide association analyses were conducted using Efficient Mixed-Model Association Expedited (EMMAX) software(version emmax-intel64-20120205), which implements the model MLM (mixed linear model) and adds kinship matrix (K) and PCA result correction to control for population structure and relatedness. This approach reduces spurious associations and improves the detection of marker–trait associations. SNP markers were determined to be significantly associated with the trait of lodging score when −log10 (*p*) = 5. Results are presented in Manhattan and Q-Q plots.

### 4.5. Candidate Gene Identification and Haplotype Analysis

A significance threshold of −log_10_ (*p*) > 5 was used in the Manhattan plot to identify associated loci. Based on the degree of LD attenuation in the soybean population, 50 kb regions upstream and downstream of the significant locus, totaling 100 kb, were defined as candidate regions. The functional annotation of genes within these regions was performed using the Soybase (https://www.SoyBase.org/, accessed 1 November 2024) in conjunction with the genome annotation file. SNPs located between the 5′UTR and 3′UTR of all genes within the candidate interval were retrieved from the resequencing dataset. Haplotype analysis was conducted using the R package “geneHapR” v1.2.4 [69], focusing on sequence variations within the coding sequence (CDS) of the target gene. Then, an annotation of SNPs located in the candidate region was conducted using ANNOVAR, Available online: https://annovar.openbioinformatics.org (accessed on 10 October 2024). This haplotype analysis exclusively included SNPs that resulted in non-synonymous mutations, frameshifts, or stop gains. In the absence of these SNP types, we selected SNPs for analysis that had a missing data frequency of less than 20%. The SNPs with a missing rate of less than 20% in the promoter regions of *Glyma.19G212700* and *Glyma.19G212800* were utilized for haplotype analysis. The same VCF file used for GWAS was employed in this analysis.

### 4.6. RNA Sequencing and Quantitative Real-Time PCR Analysis

Based on the results of multiple years of field evaluations, lodging-resistant cultivar Tiedou 113 and the lodging-susceptible cultivar Dandou 12 were selected, setting two planting densities of 300,000 and 150,000 plants/ha, respectively. Samples were taken at the full pod stage (R4), with 1 cm sections from the middle of the 1–5 nodes of soybean stems mixed, and 5 plant samples collected for each treatment to form biological samples. All samples were rapidly frozen with liquid nitrogen and then stored at −80 °C for RNA extraction. Transcriptome sequencing was entrusted to Qingdao Biomarker Bio Company. We used Fold Change ≥ 2 and FDR < 0.01 as the screening criteria for differential genes (DEGs) and performed data analysis using the bioinformatics analysis process provided by the BMK Cloud platform (www.biocloud.net, accessed on 20 February 2024). Candidate genes were analyzed by quantitative real-time PCR (qRT-PCR) based on RNAseq results. All primers listed in Table S4 were designed using the Primer-BLAST tool from the National Center for Biotechnology Information (NCBI) (https://www.ncbi.nlm.nih.gov/tools/primer-blast/, accessed on 10 January 2024). Actin was used as a reference gene. Relative expression levels were calculated using the 2^−ΔΔCt^ method. Each sample was analyzed with three independent biological replicates.

### 4.7. Statistical Analysis

IBM SPSS Statistics26.0 software (SPSS, Inc., Chicago, IL, USA) was used to calculate the mean, standard deviation (SD), and coefficient of variation (CV = standard deviation/mean) of each agronomic trait. The BLUP value of each trait and heritability (*H*^2^) were calculated using the lme4r R package v1.1-37. For multi-year multi-location data, the phenotypic value was taken as the observed value, and the location, year, and sample were taken as random factors. Local Manhattan plots and linkage disequilibrium (LD) heatmaps for SNPs surrounding peak loci were generated using the R package LDBlockShow v1.40. The gene expression patterns in soybean tissues were supported by publicly available data from phytozome platform (https://phytozome-next.jgi.doe.gov, accessed on 5 November 2024). An analysis of variance (ANOVA) in IBM SPSS Statistics 27 was used to assess significant differences in lodging resistance indices among different haplotypes and the qRT-PCR results of distinct genes with visualizations created in GraphPad Prism (v10.0.1).

## 5. Conclusions

In this study, 9,400,987 high-quality SNP markers were used to conduct genome-wide association analysis on 338 soybean germplasm resources using the MLM model, and a total of 13 significantly associated stable SNP loci were screened under two planting densities. Seven candidate genes related to lodging score were identified, among which the Hap2 haplotype of the *Glyma.19g212800* (*SUS3*) genes have important utilization value for soybean lodging resistance breeding.

## 6. Patents

The research grants [2021YFD1201102] were from the National Key Research and Development Plan of Ministry of Science and Technology.

## Figures and Tables

**Table 1:** Statistical and differential analysis of lodging traits in 338 soybean accessions across varying environmental conditions.

Trait	Environments	Density (×10^4^ Plants/ha)	Range	Mean	SD	CV%	H^2^
Lodging Score	DL2023	30	1.00–5.00	3.919	0.944	24.13	0.55
	SH2023	30	1.00–5.00	4.398	0.642	14.61	
	HN2023	30	1.00–5.00	3.588	1.014	28.22	
	SH2022	30	1.10–5.00	3.502	1.037	29.64	
	Mean	30	1.05–5.00	3.852	0.909	24.10	
	DL2023	15	1.00–5.00	3.612	1.158	32.15	0.52
	SH2023	15	1.00–5.00	4.219	0.781	18.51	
	HN2023	15	1.00–5.00	3.131	1.058	33.86	
	SH2022	15	1.00–5.00	3.073	1.159	37.70	
	Mean	15	1.00–5.00	3.509	1.039	30.59	

**Table 2:** SNP loci significantly associated with lodging traits detected at different planting densities.

Chr.	Peak Loci	PEAK Position	−log 10 (*p*)	PVE (%)	MAF
1	Chr01:39325847	39,325,847	5.31–6.03	6.03–6.91	0.022
1	Chr01:44922215	44,922,215	5.76–5.21	5.90–6.60	0.034
2	Chr02:2604732	2,604,732	5.53–5.54	6.31–6.32	0.019
2	Chr02:15458372	15,458,372	6.48–6.81	6.66–7.47	0.028
3	Chr03.:35831309	35,831,309	5.31–5.62	6.01–6.42	0.092
3	Chr03:36451125	36,451,125	6.30–7.19	7.04–8.35	0.158
4	Chr04:47442227	47,442,227	5.31–5.69	6.04–6.50	0.021
7	Chr07:20616713	20,616,713	5.32–5.37	6.03–6.11	0.015
8	Chr08:30754555	3,075,4555	5.99–6.51	6.86–7.57	0.024
8	Chr08:32270717	3,2270,717	5.14–6.12	5.81–7.04	0.013
9	Chr09:35877303	35,877,303	5.52–6.26	6.29–7.19	0.021
10	Chr10:5376997	5,376,997	5.45–6.27	6.19–7.22	0.075
11	Chr11:3581424	3,581,424	5.61–6.85	6.39–7.94	0.050
14	Chr1.:326408	326,408	6.21–6.22	7.13–7.14	0.411
14	Chr14:617529	617,529	5.21–5.55	5.90–6.33	0.450
15	Chr15:49852803	49,852,803	5.59–5.75	6.38–6.61	0.058
18	Chr18:15099487	15,099,487	5.46–5.60	5.73–6.22	0.040
19	Chr19:44477717	44,477,717	5.84–7.09	6.69–8.20	0.432
19	Chr19:46692661	46,692,661	7.85–9.45	9.16–10.99	0.327
20	Chr20:7716540	7,716,540	5.28–6.45	5.98–7.44	0.120

**Table 3:** Predicted candidate genes on chromosome 19 and functional annotations.

SNP Location	Chr.	Location	Gene ID	Gene Annotation
Chr19.:44477717	Chr19	45,769,759	*Glyma.19G200800*	Nuclear factor Y, subunit A10
Chr19.:46692661	Chr19	45,999,764	*Glyma.19G203400*	Encodes glutamate carboxypeptidase
Chr19.:46692661	Chr19	46,629,986	*Glyma.19G212700*	Glycosyl hydrolase 9B13
Chr19.:46692661	Chr19	46,635,492	*Glyma.19G212800*	Sucrose synthase 3
Chr19.:46692661	Chr19	46,856,657	*Glyma.19G215500*	Beta-xylosidase 2
Chr19.:46692661	Chr19	46,960,586	*Glyma.19G216600*	SET domain-containing protein
Chr19.:46692661	Chr19	47,352,760	*Glyma.19G221700*	WRKY family transcription factor

## Data Availability

All data presented in this manuscript are included in the Supplementary Materials.

## Supplementary Materials

The following supporting information can be downloaded at https://www.mdpi.com/article/10.3390/ijms26094446/s1.
