# Supplementary material for: Genome-Wide Association Study to Identify Soybean Lodging Resistance Loci and Candidate Genes
Source: Int J Mol Sci. 2025 May 7;26(9):4446. doi: 10.3390/ijms26094446 (PMC12072681; doi:10.3390/ijms26094446)
Supplement: Supplementary file 1 [file ijms-26-04446-s001.zip › Supplemental figure 1.pdf]

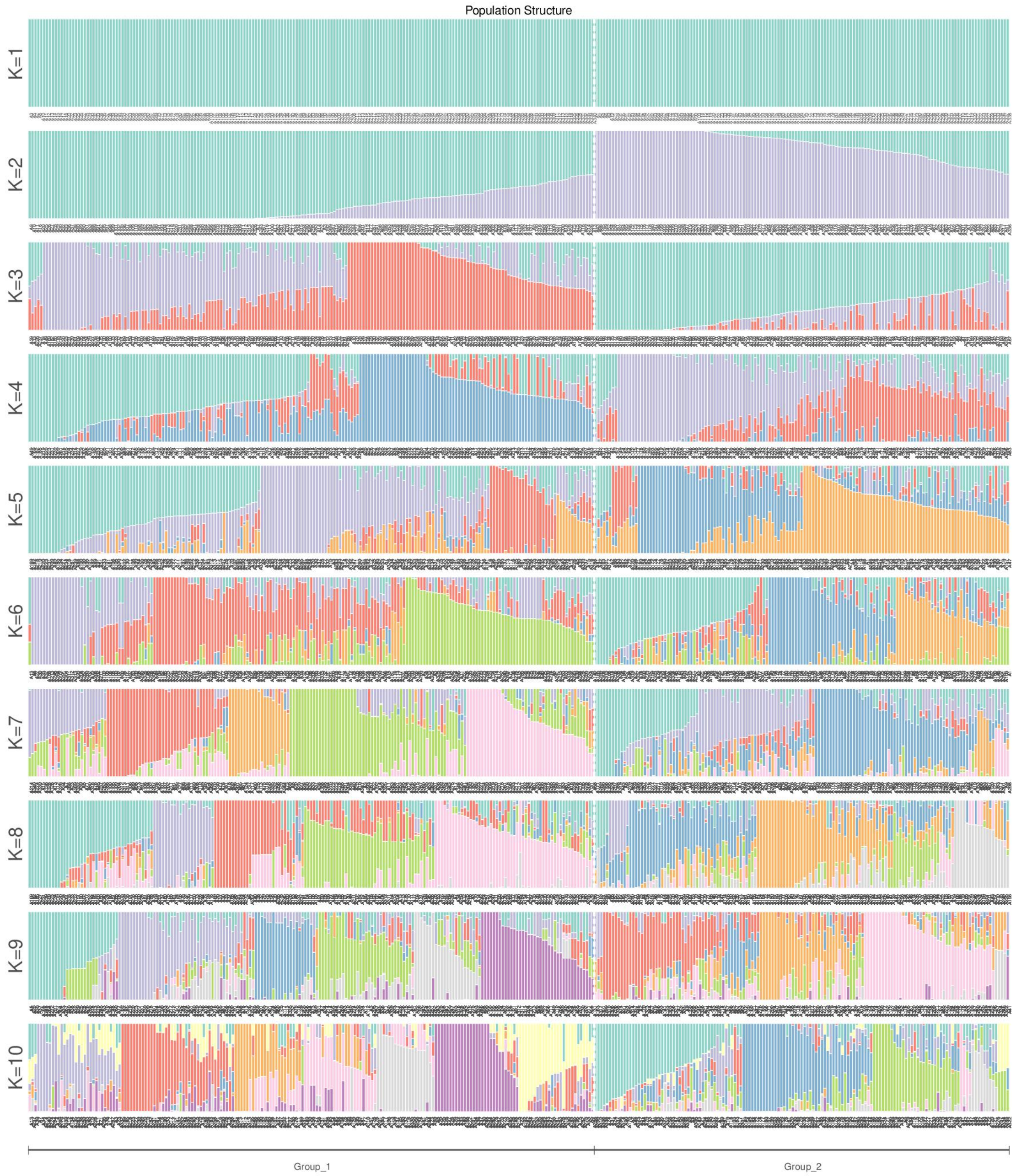

Figure S1. A bar plot diagram showing the results of clustering analysis when the number of subgroups (K) =1-10.
